# Adjustment of Clasps

**Published:** 1850-01

**Authors:** P. H. Austen

**Affiliations:** 38 N. Charles-st., Baltimore.


### ARTICLE V.

### *Adjustment of Clasps.*

MESSRS. EDITORS:

IN my last communication, I presented some objections to the use of clasps: I now offer to your readers a plan for their accurate adjustment, where their use is deemed advisable. As, when presenting those objections, I did not wholly condemn their employment; so now, in offering this plan, I do not advocate, in every case, their adoption.

It is unjust to base the rejection of any practice upon the evils attending its abuse. Thousands of teeth are daily injured by the file in the hands of ignorant pretenders; and gold fillings—so called—drop out by scores, leaving the teeth in worse condition than before. Yet the file is invaluable, and gold foil the only good means for the arrest of dental decay. If a filed surface, after the most careful finish given to it on the part of the operator, and the most scrupulous cleanliness on the part of the patient, shows greater tendency to decay than other surfaces of the teeth in the same mouth, there lies a valid objection to the use of this tool—not otherwise. If the decomposed bone be completely removed, the cavity properly shaped, the gold well introduced and condensed, and its surface rightly finished, dental art has done all that can be done to stay the progress of caries at that point. The gold is not at fault because the operator is unskilful in its use; nor the file objectionable because some handle it as if it were a horse-rasp.

My objections to clasps hold good, however accurately they may be adapted. The evil is aggravated by malpractice, but no degree of skill can altogether remove it. Clasps which are badly fitted give more ready lodgment to decaying agents, but no accuracy of adaptation can exclude them entirely. Clasps may, by bad adjustment, bring an unnecessary strain upon the tooth; but in some cases—as where the entire weight of a heavy piece is in front of the clasped teeth—this strain is unavoidable; and in all cases there is some degree of traction, else how could the clasp aid in the retention of the piece. The simple contact of metal, when brought into close and constant contact with the tooth, near its neck, is, to a greater or less extent, irritating. For this reason I never allow the plate to approach within less than half a line of the tooth; for this reason I place the clasps as far from the gum as is consistent with a firm and secure hold; and when forced, from the peculiar shape of the tooth, to encircle its neck, I feel that I have imparted to my work an element of ultimate failure.

I admit, with Dr. C. T. Cushman, that clasps are, with our present knowledge, indispensable; doubt, with him, whether they can ever be wholly dispensed with; and agree that their perfect adaptation modifies my objections; but I cannot think that these objections "vanish" before even a "*perfect*" adjustment. They were made, assuming the work to be done in the best possible manner, and they were based upon the action of physiological laws, which no mechanical ingenuity can subvert.

But though compelled, in this opinion, to differ from Dr. Cushman, I cannot omit expressing the pleasure which his article gave. So few act out the principle that "what is worth doing at all is worth doing well;" so many seek the plan which shall give least trouble, instead of searching out that which shall best accomplish the given end; so many follow a routine, without thought of improving a confessedly imperfect practice—that it gives pleasure to meet one who, in the attainment of a desired object, counts not the cost of a little additional trouble.

To those who, in a complicated piece of dental work, ask only three visits from the patient, lest that patient should weary with frequent coming; to those who, knowing a better plan, adjust clasps by the plaster cast alone, because it is more expeditious and less troublesome—to such, the "temporary fastenings" of Dr. Fogle, and the method which is here offered, will seem uselessly tedious. Not so, however, to the sincere inquirer after correct and accurate practice. It is to such that Dr. Cushman's article appeals, and to such that these remarks are directed. We confidently assert that there is no one element more essential to successful practice in mechanical dentistry than scrupulous exactness.

To say of clasps, as very commonly applied, that they are "adapted," is a perversion of language. It is customary with many to trust to the plaster cast without trying the piece in the mouth previously to soldering. An accurate adjustment is, under such practice, impossible. Others finish the adjustment in the mouth, adopting various measures to insure success. The method of Dr. Fogle secures, perhaps, greater accuracy than any other, with one exception.

Dr. Cushman has offered us a plan which he "considers so valuable that he does not venture, in the simplest cases," to use any other. We beg to return the favor, and present to Dr. Cushman a plan which we regard so highly, that under no possible circumstances would we dispense with its use. The profession is indebted to Mr. Lester Noble, of Longmeadow, Mass., for this valuable suggestion.

### *Mr. Noble's Method.*

The first step is—after the plate has been accurately fitted and filed away to the distance of at least half a line from the neck of every tooth—to fit the clasps to the teeth. It is foreign to our present purpose to speak of the variations in length, width and thickness which particular cases demand. Suffice it to say, that the clasp should fit closely every part of the tooth which it touches; must take a firm hold, even should it be necessary for this end to carry it close to the gum; must have proper width and thickness to give strength, and be sufficiently alloyed to give the requisite elasticity.

The original wax impression taken for the plate, is never relied upon for the model of the tooth to be clasped, because, in the great majority of cases, the wax is dragged at the very point where accuracy is required.

A separate impression in wax is therefore to be taken of each tooth to be clasped. This may, in a few moments, be done after the larger impression is taken, and consequently does not require a separate visit from the patient. The wax should be protected from the lateral compression of the fingers by a narrow circle of brass or silver, about one-half or three-fourths of an inch in diameter; these may be variously shaped to suit different positions. It is evident that any ooth, however irregularly placed, may be thus separately taken with the utmost accuracy.

This impression is filled, as usual, with plaster, and when preparing the larger metallic casts—always done in sand—a zinc duplicate of this tooth is readily procured. The advantage of the metallic tooth is, that the clasp can be fitted around it without danger of altering its shape, as happens with the plaster from frequent trial of the gold band. It also allows the use of other tools than the pliers in the shaping of difficult clasps. Mr. Cushman's plan of taking a pattern in sheet lead, by which to cut out the gold, is always observed. When the clasp is thus accurately fitted over the metallic tooth, in just such position upon that tooth as it is designed to hold in the mouth, it may then be tried upon the plaster tooth, and if it fit there it is ready to be tried upon the natural tooth. With proper care it can be made to fit every part of the tooth which it touches so closely as to exclude every thing except the fluids of the mouth.

You are now prepared, at this, your patient's second visit—after assuring yourself, first of the adaptation of the plate, and then of each clasp severally—to obtain the relative position of the parts, *in situ.* Here lies the peculiar excellence of Mr. Noble's method. Wax, so commonly used, is, from its soft and pliant nature, very liable to alter its shape upon withdrawal. So apt is this to happen, that perfect accuracy by this method is to be regarded as an exception and an accident. Unfortunately, the wax gives by its appearance no evidence of this change, and we learn it when correction is too late. Mr. Fogle's temporary fastenings are designed to obviate this difficulty—how perfectly, we are unable, from want of experience, to say. Mr. Noble completely obviates it by the use of plaster.

Let the clasp bind upon the tooth only with sufficient firmness to keep it in its proper place. Then mix a small quantity of plaster from a lot which, by previous trial, you find requires from six to ten minutes to set; put it upon a piece of paper or sheet lead about an inch square, and just before it begins to harden, introduce it into the mouth upon the forefinger, pressing it into gentle contact with a portion of the plate and about one-half of the clasp. It must be held there for from three to six minutes, until it is sufficiently hard to break with a sharp fracture; this point you can determine by examining the plaster left in your bowl. The plaster must then be withdrawn. Sometimes plate, clasp and plaster will be brought away together; or the plaster and clasp together, leaving the plate; or the plaster will separate, leaving both clasp and plate in the mouth. Should the plaster by any accident break, it can readily be united at the point of the fracture, without in the least altering its shape—one great advantage over wax. If the plaster adheres to the plate on withdrawal from the mouth, it must then be carefully detached, the plate replaced, and the same process repeated for the second clasp.

Several precautions are necessary. If the clasp bind too tightly around the tooth, its ends will, when removed, spring together, and thus it will not exactly fill the original impression made in the plaster. If the part of the clasp which you design to cover with plaster be so regular in shape as to make its adjustment, when out of the mouth, uncertain, mark it with a file or by a small point of solder; this will be copied in the plaster and remove all doubt as to its definite position. If the plaster be extended over some part of the edge of the plate, it will, in the absence of any marked irregularities of surface, give a better guide for its re-adaptation. Lastly, if the plaster cover too much of the clasped tooth, it will be more liable to break on being withdrawn.

Take now the clasps, place them each in their separate impressions in the pieces of plaster, securing them if necessary by a small piece of softened wax. Place one end of your plate in its corresponding bed in one of the plaster pieces. If proper care has been used, both clasp and plate will fit into the plaster with unerring accuracy, and of course hold the precise relation as when in the mouth. While in this position, cover the clasp and part of the plate on its upper surface with fresh plaster—or plaster and sand—and when this has hardened, remove the first plaster—just as in other cases you would remove the wax—preparatory to soldering.

I never unite the clasp to the plate to the extent of more than two, or at most three lines, and I place the point of attachment as near to the center of the clasp as possible. It is thus more yielding and elastic than when bound by solder to its very extremity, and the parts on either side of the point of attachment grasp the tooth with equal pressure. The strength of the union is, under this plan, gained more by the thickness than the width of the solder: this thickness is secured with greater certainty by laying a prism-shaped piece of metal at the point of union, and upon this placing the solder. The extent to which you wish the solder to flow over the plate, may be very exactly defined by the use of whiting. But these remarks apply to any mode of attaching clasps: we shall, therefore, not extend them, as our present purpose is simply to call attention—first to the importance of taking a separate impression of the tooth to be clasped; secondly, to the substitution of plaster for wax in copying the relative position of clasp and plate.

We present the above plan of Mr. Noble to the careful, pains-taking and conscientious dentist, convinced that if he will once give it an honest trial, he will adopt it as his invariable practice. It requires patience and delicate manipulation, but so does all dental work, and he who either cannot or will not comply with this requisition, is unqualified for the proper discharge of his professional duties. P. H. AUSTEN.

38 N. CHARLES-ST., BALTIMORE.

---

### ARTICLE VI.

[From the Dental News Letter.]

